# Super-Resolution Localization Microscopy of γ-H2AX and Heterochromatin after Folate Deficiency

**DOI:** 10.3390/ijms18081726

**Published:** 2017-08-08

**Authors:** Margund Bach, Claudia Savini, Matthias Krufczik, Christoph Cremer, Frank Röesl, Michael Hausmann

**Affiliations:** 1 Kirchhoff-Institute for Physics, Heidelberg University, Im Neuenheimer Feld 227, Heidelberg 69120, Germany; mbach@kip.uni-heidelberg.de (M.B.); krufczik@kip.uni-heidelberg.de (M.K.); C.Cremer@imb-mainz.de (C.C.); 2 German Cancer Research Center (DKFZ), Im Neuenheimer Feld 280, Heidelberg 69120, Germany; claudia.savini2@gmail.co (C.S.); f.roesl@dkfz-heidelberg.de (F.R.); 3 Institute for Molecular Biology, Ackermannweg 4, Mainz 55128, Germany

**Keywords:** single molecule localization microscopy, folate deficiency, γ-H2AX cluster formation, chromatin re-organization

## Abstract

Folate is an essential water-soluble vitamin in food and nutrition supplements. As a one-carbon source, it is involved in many central regulatory processes, such as DNA, RNA, and protein methylation as well as DNA synthesis and repair. Deficiency in folate is considered to be associated with an increased incidence of several malignancies, including cervical cancer that is etiologically linked to an infection with “high-risk” human papilloma viruses (HPV). However, it is still not known how a recommended increase in dietary folate after its deprivation affects the physiological status of cells. To study the impact of folate depletion and its subsequent reconstitution in single cells, we used quantitative chromatin conformation measurements obtained by super-resolution fluorescence microscopy, i.e., single molecule localization microscopy (SMLM). As a read-out, we examined the levels and the (re)positioning of γ-H2AX tags and histone H3K9me3 heterochromatin tags after immunostaining in three-dimensional (3D)-conserved cell nuclei. As model, we used HPV16 positive immortalized human keratinocytes that were cultivated under normal, folate deficient, and reconstituted conditions for different periods of time. The results were compared to cells continuously cultivated in standard folate medium. After 13 weeks in low folate, an increase in the phosphorylation of the histone H2AX was noted, indicative of an accumulation of DNA double strand breaks. DNA repair activity represented by the formation of those γ-H2AX clusters was maintained during the following 15 weeks of examination. However, the clustered arrangements of tags appeared to relax in a time-dependent manner. Parallel to the repair activity, the chromatin methylation activity increased as detected by H3K9me3 tags. The progress of DNA double strand repair was accompanied by a reduction of the detected nucleosome density around the γ-H2AX clusters, suggesting a shift from hetero- to euchromatin to allow access to the repair machinery. In conclusion, these data demonstrated a folate-dependent repair activity and chromatin re-organization on the SMLM nanoscale level. This offers new opportunities to further investigate folate-induced chromatin re-organization and the associated mechanisms.

## 1. Introduction

Folate is a water-soluble B vitamin implicated in one-carbon metabolism, playing an important role in DNA nucleotide synthesis and methylation reactions [1]. The DNA methylation machinery induces histone modifications and chromatin remodeling activities that act cooperatively in transcription regulation [2,3]. Several studies have highlighted the association between folate deficiency and an increasing incidence of certain types of malignancies, including human papilloma virus (HPV)-induced cervical cancer [4,5,6,7,8,9]. The mechanisms of how the folate status, especially a low folate status, contributes to these diseases remain unclear. Conformational changes of chromatin can be induced by DNA methylation modifications that are in turn also regulated by folate [10]. Moreover, depending on the cell type, changes of the folate status can either lead to hypo- or to hyper-methylation, both resulting in non-physiological effects on a cellular system [11].

Folate is also involved in nucleotide synthesis and repair by converting uracil to thymine. In the case of folate deficiency, uracil is incorporated into DNA during replication. This means that inadequate folate availability may lead to mutagenic effects and trigger attempts to repair [12]. Therefore, the excision of uracil from DNA can create DNA breaks that are increasingly accumulated in case of a constantly limited nucleotide pool [13]. Folate deficiency can lead to elevated DNA damage and replication stress per se, and can also have an additive effect on oncogene-induced DNA instability [14,15], or can destabilize telomeres by affecting the G-quadruplex structure of the DNA [10].

High folate concentrations resulting in high global DNA methylation have been shown to reduce cancer risk [16,17]. However, there is no objective recommendation of the required folate concentration for healthy outcomes. Conflicting results illustrate the complexity of such processes, and it remains unclear whether folate deficiency is predisposing for carcinogenesis, promoting cancer, or even preventing cancer [11].

Considering the complexity of the cell metabolism, it is obvious that a linear correlation or dose response of folate, DNA methylation, and structural chromatin organization cannot be applied. The present study was aimed at investigating the impact of in vitro folate depletion/reconstitution on DNA stability and chromatin organization by super-resolution fluorescence microscopy [18]. In particular, we explored for the first time the effect of folate deficiency on chromatin structure and its condensation status by single molecule localization microscopy of the fluorescent tags of γ-H2AX chromatin phosphorylation sites and of H3K9me3 methylation. This histone marker was selected since H3K9me3 is involved in heterochromatin formation [19]. We focused our interest on structural changes that facilitate the accessibility of damaged sites to the DNA repair machinery. Moreover, the reversibility of these changes was explored by analysing different conditions of folate availability. As cellular model, human foreskin keratinocytes immortalized by expressing E6 and E7 oncoproteins of human papillomavirus type 16 were used [20,21].

H2AX is phosphorylated (referred as γ-H2AX) [22] in the vicinity of DNA double strand breaks (DSBs), where also other proteins of the repair machinery accumulate to form microscopically visible repair foci. Since γ-H2AX is a sensitive and early indicator of DSBs in vitro and in vivo [23], it is an accepted marker to measure for DNA damage [24]. Several studies showed that the γ-H2AX background level varies depending on cell type and cell status [25,26].

The chromatin threads of individual chromosomes are hierarchically organized, and the higher order nuclear and chromatin architectures are closely related to genome functions, cell type, and differentiation state [27,28]. As a result, different condensed chromatin domains exist: the transcriptionally active euchromatin and the largely inactive, tightly compacted heterochromatin [29,30]. Cells react by a complex DNA damage response that triggers DSB signaling and repair processes accompanied by systematic chromatin re-arrangements and heterochromatin de-condensation for the access of repair-associated proteins and a sub-diffusive movement of DNA break ends [31,32,33,34].

Here, we present the application of single molecule localization microscopy (SMLM) [35] to study chromatin organization, H2AX phosphorylation, and foci formation around DNA double strand break regions. SMLM circumvents the Abbe–Rayleigh boundary conditions of diffraction [36] normally causing resolution limits. The advantage of SMLM is that the data of a molecule loci matrix can be obtained without any image and image processing. The raw data of the coordinates can either be used for structure evaluation or for the calculation of an image as a secondary result. Thus, the image can also encode structural parameters as point densities or a cluster formation that has been calculated before image construction. In that way, images showing an effective optical resolution down to the order of 10 nm can be accomplished [35] (defining resolution as the minimum distance between two points that can precisely be measured). SMLM is based on the fundamental concept of the optical isolation of objects by different spectral signatures [36], e.g., by constant differences in the absorption/emission spectrum [37], differences in the fluorescence life time, or by the detection of fluorophores that can be switched between two different spectral states, e.g., on and off [38,39,40] to achieve a spatial separation of single signals. From a reversible dark state, fluorescent molecules stochastically return to the emission state. The centre-of-mass (barycentre) of an Airy disc obtained as a point source image approximates the location of the emitting molecule. This allows the determination of object positions and spatial distances between labeled points [41,42], also if their distance is considerably smaller than the light wavelength used for fluorescence excitation. From all acquired coordinates of fluorescent molecules and the precision of loci detection, a “pointillist”, super-resolution image can be reconstructed in which the effective resolution depends on the signal to background ratio of the specimen. So far, different applications have demonstrated the power of localization microscopy for biological measurements, e.g., in analyses of chromatin in cell nuclei [31,34,43].

## 2. Results

As an in vitro model, primary human foreskin keratinocytes (HFK) were immortalized by expressing human papillomavirus type 16 (HPV16) E6 and E7 oncogenes (HFK16E6E7 cells). These cells were cultured with a standard amount of folate present in the culture medium. The concentration of folate was 4.5 µM (folate control condition = FC). Folate deficiency was mimicked by using folate-free culture medium (folate deficiency condition = FD). The only contribution of folate was provided by fetal bovine serum (FBS), and was estimated to be equal to 0.002 µM. Cells were propagated in cultures under FC and FD conditions for more than 12 weeks to ensure a stable in vitro cellular phenotype [21].

**Figure 1:**
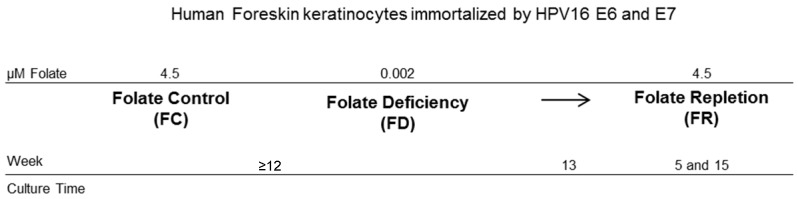
Schematic overview of the experimental cellular model and cultivation time course.

Thirteen weeks after cultivation start, aliquots of the specimens were fixed and subjected to two-colour SMLM. These samples are designated “FC and FD”, respectively. Reversibility of depletion was explored by restoring a culture medium with a standard amount of folate for 5 or 15 weeks after a period of 13 weeks of folate deficiency. These samples are denoted as “FR5 and FR15”. A schematic overview is showed in Figure 1.

**Figure 2:**
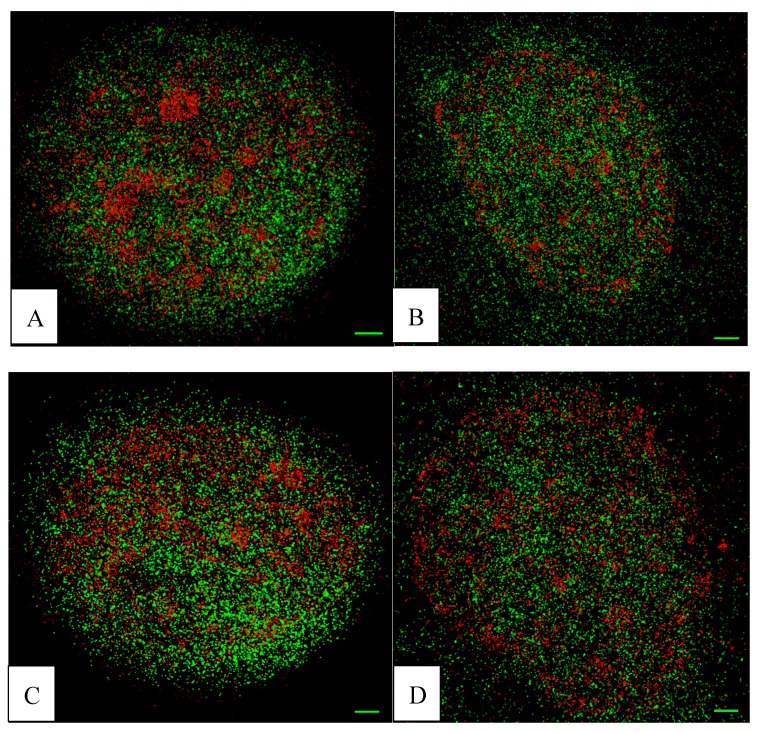
Examples of pointillist image sections obtained by reconstruction from the “orte-matrix” of the cell nuclei. Two colour planes are merged. Each point represents the position of a fluorescent tag (red: H3K9me3; green: γ-H2AX). Further counterstaining was omitted. (**A**) FC after 13 weeks cultivation; typically heterochromatin shows high and less-condensed regions, and γ-H2AX is dispersed over the whole nucleus; (**B**) FD (=13 weeks folate depletion); the γ-H2AX distribution seems to be dispersed without cluster formation, whereas the heterochromatin appears to be less condensed; (**C**) FR5 (=5 weeks folate repletion); there seems to appear a recovery of dense heterochromatin regions with γ-H2AX dispersed but also clustered; (**D**) FR15 (=13 weeks folate depletion and 15 weeks folate repletion); the heterochromatin seems to be clustered, but the number of γ-H2AX signals seems to be reduced. For further details see quantitative results; scale-bar = 1 µm.

In two measurement campaigns, up to 40 cell nuclei were recorded by SMLM and the detected loci matrices (orte-matrix) were evaluated according to different parameters. SMLM is a measuring technique that does not need any imaging procedures for data evaluation, since the data can be obtained without image. The coordinate matrix can be scaled by the absolute size values of the camera pixels. Based on this knowledge, distances can be given in absolute values and pointillist images can artificially be prepared as a demonstrator of the detected structures. In Figure 2, examples of such pointillist images prepared from the “orte-matrix” are shown. Each point represents the position of a fluorescent molecule (red H3K9me3; green γ-H2AX).

**Figure 3:**
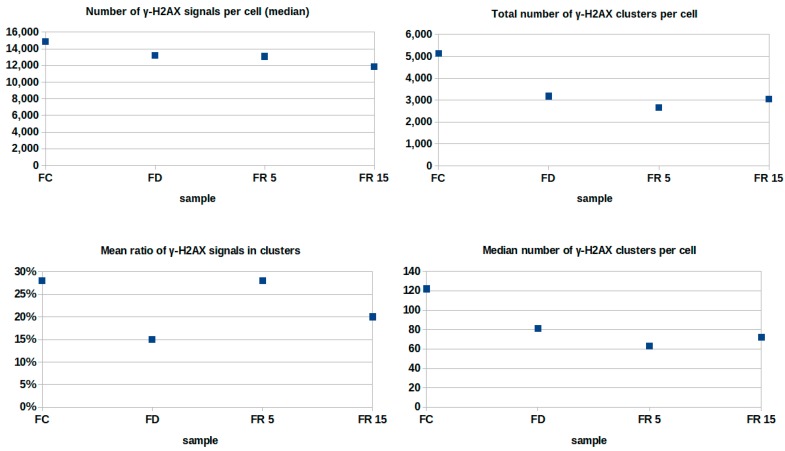
Quantitative evaluation (absolute numbers and ratio in %) of γ-H2AX signals for four different preparation conditions.

Since the recorded signals are always taken from a central optical section of a cell nucleus within a region of interest (RoI) containing one nucleus completely, absolute numbers of points are representative and can be compared directly. In Figure 3, the signals obtained from γ-H2AX labeling are evaluated for the different specimens mentioned above. The median number of signals was lower for FD conditions than for FC conditions. An increase occurred in FD and was maintained for 5 weeks after folate re-supplementation (FR5); but subsequently reduced after 15 weeks (FR15). Defining cluster conditions (minimum five neighbouring points within a radius of 100 nm), between 10% and 33% of the detected signals were found on average in clusters. Under FD conditions, the cluster formation dropped down to 10% after 13 weeks. After 5 weeks in normal folate medium (FR5), the cluster formation was the same as for cells having been maintained in FC conditions continuously. At FR15, the cluster formation decreased, indicating a relaxation of the repair foci which may be due to reduced repair activity. We also counted the total number of detected clusters and the median number of clusters per cell for the different conditions. These numbers were about 60% of the FC values for FD and FR5 or FR15. In addition, the numbers appeared relatively constant at the FD level although the cells remained in cultivation conditions with normal folate concentrations over a period of 15 weeks.

**Figure 4:**
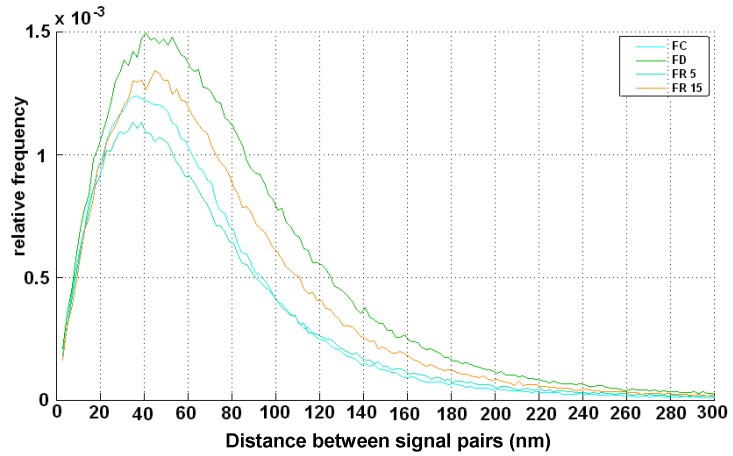
Relative frequency of signal-pair distances (normalized over all distances) in γ-H2AX clusters for four different preparation conditions.

Figure 4 shows the normalized frequency of distances between signal pairs within clusters. For FC conditions, these show a maximum frequency at 40 nm point distance. Under FD conditions, the clusters were more relaxed (increased width of the peak) with a maximum frequency at 50 nm point distance (FD). For FR5, a strong compaction of the clusters occurred compatible to the situation of FC. This indicates a recovery of the DNA repair processes, since the formation of compacted clusters is an established signature for activated DNA double strand repair. At FR15, the clusters seem to relax again, which well agrees to the other data shown above.

**Figure 5:**
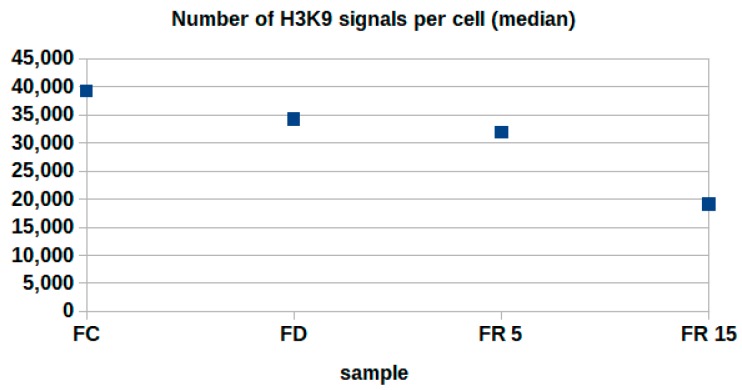
Median numbers of H3K9me3 signals for 4 different preparation conditions.

In the second colour plain, the position coordinates of the labeling tags of H3K9me3 methylation sites were recorded. In Figure 5, the median numbers of these signals are shown. For FD conditions, there is a significant reduction of methylation sites compared to FC control cells. For FR5 conditions, the number is similar to the value of FD. This value drops down again for FR15. However, the latter might not exclusively be due to a reduced methylation. It seems to result from a compaction effect of heterochromatin after successful repair, which might result in a lower accessibility for the antibodies due to steric hindering.

**Figure 6:**
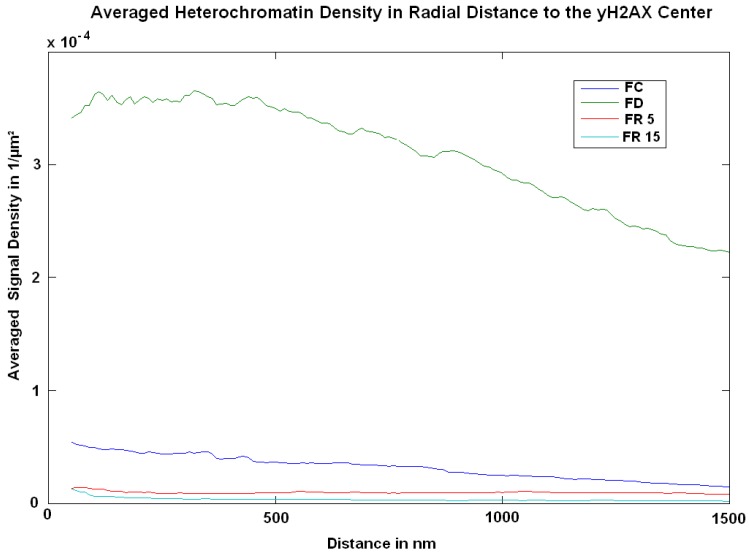
Averaged density of heterochromatin signals (H3K9me3) around γ-H2AX clusters. The data show the point density in 1/µm^2^ vs. the distance from the cluster barycentre. Comparison of FC and FD conditions with FR5 and FR15 conditions.

In order to verify the effects suggested by the analysis of the signal numbers, the clusters were closed by appropriate image calculation procedures and the barycentre were determined. These barycentre were used as the centre points of increasing circle shells with the size of 20 nm. The heterochromatin density in these shells was computed (Figure 6). Comparing with the 13 weeks values of FC, FD shows a considerably increased density of H3K9me3 tags around the γ-H2AX clusters. The curves indicate higher values outside the clusters. For FR5 and FR15, these densities were considerably lower, even lower than the control (FC), and did not show any fluctuations, indicating normally compacted heterochromatin.

## 3. Discussion

Recent research has shown that the genome architecture is not random, neither on the micro- nor on the nano-scale, as represented by the arrangement of chromatin and protein complexes in a chromatin environment [27,28,30]. In fact, there exists a close correlation between genome architecture and functional activities depending on environmental stress. Thus, it is not surprising that the cellular damage response is associated with chromatin and protein conformation changes on the nano-scale.

In this report we aimed to investigate the impact of in vitro folate deficiency on DNA stability by super-resolution fluorescence microscopy [18]. To our best knowledge, these investigations are the first applications of SMLM over such a long term culture and serious impact on cell repair functioning. In particular, the effect of folate deficiency on chromatin structure and γ-H2AX organization was shown. This study did not intend to provide conclusive results related to mechanisms induced by folate deficiency, but rather to highlight the potentials of SMLM.

The fluorescent tags of γ-H2AX chromatin phosphorylation sites and of H3K9me3 methylation sites showed characteristic organizational changes upon folate deficiency and re-supplementation. γ-H2AX is a sensitive and early indicator of DSBs in vitro and in vivo [23]. Associated with H2AX phosphorylation, folate deficiency has an impact on H3 methylation, which is an indicator for heterochromatin. Our data verify in a reasonable way conformation changes as detected by counting and distance evaluation of the appropriate fluorescent tags. Some variation detected may relate to different cycle stages of the cells measured at random.

In contrast to our experiments presented here, DSBs induced by ionizing radiation show completely different dynamics of H2AX phosphorylation in their neighbourhood. While cluster formation is occurring in the early 30 min after DSB induction, γ-H2AX foci are relaxing during the next few hours after finalizing the repair processes [44]. This difference in cluster formation dynamics may either indicate that the repair processes activated in the folate recovery experiments may be different from those occurring after ionizing radiation damage. Conversely, this long lasting (over weeks) existing γ-H2AX formation may indicate that DSBs induced over weeks of folate deficiency could not further be repaired in the recovery phase.

During folate deficiency (FD), chromatin relaxed as compared to FC due to the accumulation of DNA damage primarily of endogenous origin and insufficient attempts of repair. Later, after folate reconstitution (FR5, FR15), chromatin re-compacts, indicating a possible decrease of DNA damage and repair due to a reconstituted nucleotide pool. However, after a further 10 weeks (FR15), we still observe a γ-H2AX cluster formation at an intermediate situation between FC and FD. This might indicate continuously existing DNA damage due to the repair process on mis-repaired sites (in correlation with an in vivo DNA ligation assay that shows an increased nucleotide loss and mis-incorporation after folate reconstitution; Savini et al., manuscript in preparation). Besides the impact of DNA damage machinery on chromatin compaction status, we cannot exclude, with the present data, a potential effect of folate variation on the methylation-dependent chromatin remodeling protein complexes.

Heterochromatin re-organization during DNA repair always requires chromatin plasticity. The repair of different cell injuries follows a set of orchestrated events as being described by the “access-repair-restore” model [45] originally developed in the context of the cellular response after UV-induced DNA damage [46]. According to this model, a considerably protracted time course has to be taken as a basis. Usually, the initial response to DNA damage includes a rapid local chromatin de-condensation in order to enable a quick access for repair proteins. Heterochromatin is refractory at the very beginning, as shown by γ-H2AX forming at the periphery of heterochromatic regions. Folate deficiency, however, may influence both the recruitment of repair proteins as well as DNA relaxation in a non-correlated way. The simple model that DNA opening favours damage response and repair might be an oversimplification. The effects on euchromatin regions should be also considered under folate depletion and repletion, since the structural constraints of tightly packed chromatin on DNA repair may be alleviated by gene activity [47]. In order to better understand such mechanisms, SMLM may be an appropriate tool for further investigations using different labeling strategies.

Beyond these possible biological mechanisms, one should also consider that the data presented here average over cluster formation and do not functionally classify clusters in a cell. Novel studies on cluster and repair foci categorization, respectively, based on SMLM and topological analyses indicate that γ-H2AX clusters could have different internal structures and therefore differences in heterochromatin compaction (Hoffmann, Krufczik et al., manuscript in preparation). Further experiments could be performed by these means, in order to better explain the reasons for differences in the repair activities. 

SMLM has the potential for further studies on the internal structure of repair clusters using further tags and novel algorithms for topological analysis. This will be subject of future publications.

## 4. Materials and Methods

### 4.1. Cell Model and Culturing

HFK16E6E7 cells are primary human foreskin keratinocytes that were immortalized by expressing Human Papillomavirus type 16 (HPV16) E6 and E7 oncogenes. Briefly, human foreskin keratinocytes were retro-virally transduced with the expression plasmid harbouring the oncogenes E6 and E7 of HPV16, under the control of Moloney murine leukemia virus promoter, as previously described in [20,21].

HFK16E6E7 cells were grown together with NIH 3T3 feeder in FAD medium that contained 3 parts Ham’s F12 (Thermo Fisher Scientific, Waltham, MA, USA), 1 part Dulbecco-modified Eagle medium (DMEM, Sigma-Aldrich, St. Louis, MO, USA), 2.5% fetal bovine serum (FBS), insulin (5 µg/mL, Sigma-Aldrich), epidermal growth factor (10 ng/mL, Sigma-Aldrich), cholera toxin (8.4 ng/mL, Sigma-Aldrich), adenine (24 µg/mL, Sigma-Aldrich), and hydrocortisone (0.4 µg/mL, Sigma-Aldrich). NIH 3T3 cell cycle arrest was induced by mitomycin C treatment (Sigma-Aldrich) in order to prepare feeder cells. Cells were cultured at 37 °C, with 5% CO_2_ and in 95% humidity. The standard FAD medium contained 4.5 µM of folate. This condition is referred as folate control (FC).

To mimic folate deficiency, HFK16E6E7 cells were also adapted to stable growth in deficient folate FAD medium with a final folate concentration of 0.002 µM, contributed from 2.5% FBS. All components of folate deficient FAD medium were similar to standard FAD medium, except for DMEM (HIMEDIA) and Ham’s F12 (PAN-Biotech, Aidenbach, Germany) that did not contain folate. These conditions are referred to as folate deficiency (FD). The cells were continuously cultivated in FD conditions for more than 12 weeks to ensure a stable in vitro model for folate deficiency. These cells were put back on standard culture conditions for further 15 weeks, similarly to FC cells. These conditions are referred as folate repletion (FR).

From the specimens prepared under different conditions, aliquots were fixed and slides were prepared at week 13 (FC, FD), after repletion of 5 weeks (FR5), and 15 weeks (FR15).

### 4.2. Slide Preparation

Cells were seeded on glass cover slides to reach 70% confluency. The cells were fixed with 4% formaldehyde for 10 min and subsequently permeabilized with 0.5% Triton X-100 for 5 min at room temperature. Non-specific binding sites were blocked with 2% bovine serum albumin (BSA), 0.1% Triton X-100, and 0.3 M glycine. Primary antibodies mouse anti-H2AX phosphorylated Ser139 (γ-H2AX; dilution 1:250; Millipor 05636) and rabbit anti-Histone H3 (tri-methyl-K9) (H3K9me3; dilution 1:1000; Abcam 8898) were applied for 1 h, followed by washes with phosphate buffered saline (PBS). As secondary antibodies, goat anti-mouse IgG Alexa-Fluor 488 (Invitrogen, Carlsbad, CA, USA) and goat anti-rabbit IgG Alexa-Fluor 594 (Invitrogen) were incubated for 45 min in the dark. Nuclei were counterstained with Hoechst 33342 (Sigma-Aldrich; 1.25 µg/mL). The slides were mounted with ProLongGold anti fade mountant (Thermo Fisher Scientific) and stored at 4 °C.

### 4.3. Single Molecule Localization Microscopy (SMLM)

For SMLM, a setup has been used [48] that combines structured illumination (not used in this application) and single molecule localization microscopy. It is an epifluorescent microscope that can change between two separate light paths, one at a high intensity density (10 kW/cm^2^) and the other at a lower one (0.2 kW/cm^2^). The former is used in the localization modus applied here. One can switch between different solid state lasers of wavelengths 488 and 568 nm. Their power was set to 200 mW, which corresponds to a power density of 10 kW/cm^2^ during SMLM data acquisition. The intensity of the laser(s) can be regulated via a neutral density filter wheel (i.e., a neutral grey filter) in up to 12 steps. Movable mirrors can switch it between the two modes.

The emitted photons are detected by a high quantum efficiency charge-coupled device (CCD) camera (Sensicam QE, PCO, Kelheim, Germany) after passing through a dichroic filter-wheel and a blocking filter-wheel (also called emission filter-wheel). The camera has a very sensitive and fast CCD-chip consisting of an array of 1376 × 1040 pixels with an area of 6.45 × 6.45 µm^2^ for each pixel. Together with the objective 100×/NA 1.4, it results in a pixel size of 64.5 × 64.5 nm^2^ for the raw data.

### 4.4. Data Acquisition and Processing

Cells were selected by visual inspection. For acquisition of wide-field images and SMLM image stacks, a region of interest (RoI) was set in such a way that the RoI covered the cell nucleus completely. After determination of a RoI, the wide-field images were taken. Once all wide-field images were acquired, the same procedure was done for SMLM data recording. The SMLM image stacks of about 1000 image frames were acquired with an integration time of 50 ms.

In order to avoid preparation effects of cells from the same specimen, only cells with a number of detected points approaching an approximate 10% range around the average point number of the same specimen were considered for further evaluation. Thus, variations only due to labeling efficiency could be avoided.

For further processing, various MATLAB-scripts were used to identify the point positions/loci and intensities of the single signals and to save them in a so-called “orte-matrix”. In order to obtain this, the program requires the conversion factor from pixels to nm. The orte-matrix consists of nine columns: (a) The amplitude of the signal in photoelectrons; (b) the lateral *y*-coordinate in nm; (c) the lateral *x*-coordinate in nm; (d,e) the measurement errors for *x* and *y* coordinates; (f,g) the standard deviations; (h) the number of photoelectrons in the signal, i.e., counts, and (i) the number of the image in which the signal is found.

Further calculations were done on the basis of the orte-matrix; for example, a frequency distribution of point distances, a cluster analysis (=minimum number of loci in a certain circle around a point), the preparation of an artificial pointillist localization image, or a nearest neighbour image. In the localization image created from the orte-matrix, the brightness of a point determines the localization precision, i.e., the brighter a point appears the more precisely it has been identified. In the nearest-neighbour image (NN-image), the brightness indicates how many signals are neighbouring the given signal in a defined surrounding.

In order to study the γ-H2AX clustering, the evaluation algorithm *autoclusters_v3* was used [49]. The algorithm identifies “cluster-points” within the localization data according to user-defined parameters. These parameters are the minimum number of fluorescence signals within a defined circular region around each blinking event and the radius of this region. An event is identified as a “cluster-point” if in the predefined radius there are at least as many points defined as minimum points. The remaining points are identified as “noise-points”. The points are then divided into clusters. If two cluster-points have a smaller distance than the given radius, they belong to one cluster. In addition, all noise-points whose distance to a cluster-point is smaller than the radius also belong to the cluster. After iterative exclusion, a cluster radius of 100 nm and a minimum number *n* = 5 neighbours within this radius were set to determine the minimum conditions for a cluster point. The cluster radius then extended from a point, which was consistent with the minimum number of *n* within the required radius. Point distance and cluster frequency distributions, cluster sizes (number of counts in a cluster), etc. were calculated.

After cluster recognition, the centre of the γ-H2AX clusters was computed with the Surveyor’s Area Formula [50]. These centres were used as the centre points of increasing circle shells with the size of 20 nm. The heterochromatin density in these shells was computed (where heterochromatin density is the number of heterochromatin points/area of the corresponding circle shell).

Note: The cluster formation is calculated from the orte-matrix and reflects the arrangement of the labeling tags quantitatively determined according to well-defined parameters. Although these clusters may reflect internal structures in repair foci, they differ in their shape and number from foci, since foci are the result of microscopic images containing, for instance, diffraction-induced image spreading.

## 5. Conclusions

In conclusion, we could show that SMLM is a valid tool for investigations in cell research and studying conformational nuclear changes on the molecular level also in cases of long-term cell culturing. By means of suitable procedures of quantitative analyses and the choice of appropriate parameters, the analysis approach can contribute to further insights and validate known dynamics of DSB-containing chromatin at the nano-scale. In addition, these ultra-resolution insights on structures and architectures could also be represented by pointillist images, and offer new perspectives to further understanding of the mechanisms driving genome conformation changes.
